# The availability of local primary care services, satisfaction with health services and self-rated health in older English adults: A population-based study

**DOI:** 10.1016/j.pmedr.2022.101786

**Published:** 2022-04-05

**Authors:** Yu-Tzu Wu, Matthew Prina, Fiona Matthews

**Affiliations:** a Population Health Sciences Institute, Newcastle University, Campus for Ageing and Vitality, Newcastle upon Tyne, UK; b Department of Health Service and Population Research, Institute of Psychiatry, Psychology and Neuroscience, King’s College London, De Crespigny Park, London, UK

**Keywords:** Primary health care, Healthy ageing, Satisfaction with health services, Self-rated health, Observational study

## Abstract

Primary care services can play an important role in addressing health inequalities and challenges of population ageing. The aim of this study is to investigate whether the availability of local primary care services can support satisfaction with health services and self-rated health in older people. This study was based on a population-based cohort study, Understanding Society: the UK Household Longitudinal Study, focusing on people aged ≥ 50 at Wave 3 (2011–2013; N = 14498) and Wave 6 (2014–2016; N = 13025) in England. Locations of primary care services, including general practitioner (GP) practices and other community health services, were identified from National Health Service Digital and linked to residential areas of the study participants. Multilevel Poisson regression modelling was used to investigate the associations between the availability of local primary care services, satisfaction with health services and self-rated health adjusting for sociodemographic factors, deprivation and urban/rural settings. Participants who had more GP practices in local areas were less likely to report dissatisfaction with health services in Wave 3 (IRR: 0.67; 95% CI: 0.52, 0.85) and Wave 6 (IRR: 0.74; 95% CI: 0.59, 0.92). No associations with self-rated health were found in both waves. These associations were similar across deprivation levels and urban/rural settings. The results suggest that increasing availability of local primary care services may improve satisfaction but not health in older people. To optimise the supportive role of primary care services in healthy ageing, future research should identify complex needs of health and social care in older people and their experience of using the services.

## 1 Introduction

Population ageing is a key issue across the world and has a substantial impact on health and social care (WHO, 2021). Compared to younger age groups, older people are more likely to experience multiple chronic conditions and severe health problems and have greater needs of health care (Nguyen et al., 2019). Primary care services in local areas, the first point of contact in the healthcare system, can play an important role in the management of chronic diseases, as they are the gate keepers of physical and psychological health conditions in older people (Age, 2021). Access to local general practitioner (GP) surgeries has also been identified as a key element of age-friendly environments and an indicator for deprivation and inequality measures in the UK (Burton and Mitchell, 2006, Uk, 2015, Uk, 2019).

In the 1970s, the ‘inverse care law’ was suggested to describe the situation that ‘the availability of good medical care tends to vary inversely with the need for it in the population served’ (Tudor, 1971). The hypothesis has been widely recognised in the UK and international societies, given that older people in deprived areas are more likely to experience poor life expectancy and health (Marmot, 2018, Nambiar and Mander, 2017). Yet some empirical evidence indicates conflicting results to this hypothesis (Adams and White, 2004, Todd et al., 2014). Based on the Index of Multiple Deprivation (IMD) 2000, areas with better access to services, which were measured by straight line distances and car travel times to the nearest GP surgery and other three services, were actually associated with higher mortality and premature limiting long-term illness particularly in urban settings (Jordan et al., 2004). Inverse relationships were also found between the distance to a GP surgery and overall deprivation scores, measured with later versions of the IMD (Uk, 2015, Uk, 2019).

The difference between the inverse care law and empirical data indicates that people living in more deprived areas had better access to primary care services but worse health. Since the role of primary care services has been highlighted in addressing health inequalities and challenges of population ageing (Royal College of General Practitioners, 2015), it is important to investigate these differences and provide empirical evidence that directly indicates how local primary care services can support health in older people and their needs. Most research used specific health conditions and mortality to define ‘the needs of healthcare’. Subjective measures such as satisfaction with local health services may provide insights into identifying those with unmet needs and potential problems of using health services.

Using a large cohort study of the general population of the UK, the aim of this study is to investigate the associations between local primary care services, satisfaction with health services and self-rated health in older people. Based on data at two time points, the analysis examined potential changes over time and explored whether the associations varied across urban/rural settings and deprivation levels.

## 2 Methods

### 2.1 Study population

Understanding Society: the UK Household Longitudinal Study (UKHLS) is an ongoing panel study of individuals in 40,000 households since 2009 (University of Essex, 2020). The study was designed to provide a representative sample of the national and regional populations in the UK accounting for socioeconomic and ethnic compositions of geographical areas. The study incorporated a large sample of general population, the participants from British Household Panel Survey (wave 2), and boost samples of the immigrant and ethnic minority. Face-to-face interviews were carried out annually by trained interviewers and included all individuals aged 10 or above living in the selected households. The main survey was given to adults aged 16 or above and a self-completion questionnaire was used to collect information on sensitive topics such as mental health, attitude to politics and gender. Participants who left the households were followed-up and new members who joined the households were included in the study. The study collected a wide range of data on education, employment, housing, lifestyle, social and family network, health and wellbeing. Written consent was obtained from all participants. More detailed information on study design and sampling methods is reported elsewhere (https://www.understandingsociety.ac.uk/documentation/mainstage).

This study focused on 14,498 people aged 50 or above at wave 3 (2011–2013) and 13,025 at wave 6 (2014–2016), which both included measures on the standard of local services. Given the availability of GP practice data, this analysis included the English participants who continuously lived at the same address since the previous waves and excluded people who only had proxy interviews (N = 1835 for wave 3; N = 2680 for wave 6) as they did not complete questions on satisfaction with local services and self-rated health.

### 2.2 Primary care services

The prescribing centre data from NHS Digital provide information on primary care services in England including GP practices, public health services, community health services, walk in centres (WIC), out of hour (OOH) practices and prescribing sites in institutional settings such as care home, hospice and prison (National Health Service Digital, 2021). This study focused on those in community settings, which were categorised into three types: GP practices, public health/community health services and WIC/OOH practices. The information on prescribing settings was not available in Wales and therefore the prescribing sites in the Welsh areas were excluded. The data on names, addresses and open/close dates were extracted and the postcode information was linked to corresponding Lower-layer Super Output Areas 2011 (LSOAs) based on National Statistics Postcode Lookup (November 2020). LSOA is a small area unit used for the UK census and included an average population of 1614 with a 95% range between 1157 and 2354 (Office for National Statistics, 2022). This study used LSOA to define a neighbourhood area where people were likely to have frequent interactions with local services. Since older adults usually spend more time in their local areas than younger people (Local Government Association, 2015), they are likely to rely on GP surgeries and other primary care services close to their homes when they experience health problems.

The availability of primary care services was measured by the numbers per LSOA, which indicated presence and amounts of primary care services in local areas. Although the IMD used indicators for ‘access’ to services, the measure actually focused on the physical availability of GP surgeries in local areas rather than the comprehensive concept of accessibility (including financial, personal and organisational barriers to use the service) (Gulliford et al., 2002). Thus, this study here used the term of ‘availability’. Using the information on open/close dates, the number of GP practices, public health/community health services and WIC/OOH practices per LSOA was calculated for year 2012 and 2015, which were the mid-year of follow-up wave 3 and 6. The measure for GP practice was categorised into three groups (0, 1, 2 or above) while the measures for public health/community health services and WIC/OOH practices were divided into two groups (0, 1 or above) due to the small numbers. Based on the LSOA codes, the measures for these primary care services were matched to residential areas of the wave 3 and 6 participants in Understanding Society. To test the potential impact of primary care services in neighbouring areas, the availability of GP practices was also estimated for LSOAs which shared a border with participants’ local LSOAs. In addition, average road distance to a GP surgery at LSOA level was extracted from the sub-domain indicator of IMD 2015 and divided into four groups: ≤0.5 km; >0.5 and ≤ 1 km; >1 and ≤ 2 km, >2 km (reference group). Since the distance measure was part of the IMD scores and could not fully match the time points of the two waves, this study only used the measure in the sensitivity analyses.

### 2.3 Outcome measures

Satisfaction with health services was embedded in the questionnaire for the standard of local services and assessed using the statement: “I’m going to read out a list of facilities and services in your local area. For each one please tell me whether you consider your local area services to be excellent, very good, fair or poor?” The rating for medical facilities was categorised into two groups: excellent/very good and fair/poor.

The measure of self-rated health has been recognised as a predictor of mortality and health conditions (Idler and Benyamini, 1997, Mavaddat et al., 2014). In Understanding Society, self-rated health was measured by the question: “In general, would you say your health is…” with four options (excellent, very good, good, fair and poor). The measure was divided into two groups: excellent/very good/good and fair/poor.

### 2.4 Covariates

Sociodemographic factors, including age, sex, social class and education, were measured in the Understanding Society surveys. Occupation-based social class was based on derived variables included in the Understanding Society data and categorised into three groups: high (professional or managerial occupations); middle (skilled occupations); and low (partly skilled, unskilled occupations or armed forces). If the participants did not have a current occupation, the last job in their working life was used to indicate their social class. The highest educational qualification was divided into four groups: higher degree, secondary school, primary school, none of above.

Two area level (LSOA) were included in this analysis. The English Indices of Multiple Deprivation (EIMD) 2015 incorporated characteristics related to poverty and socioeconomic disadvantage including income, employment, education and training, health and disability, barriers to housing and services, the living environment and crime (UK government, 2015). The measure was divided into quintiles among all 32,844 LSOA units for England. The first quintile (Q1) represents 20% of the most deprived areas in the country. The 2011 Census Rural Urban Classification (https://www.gov.uk/government/statistics/2011-rural-urban-classification) was used to derive four types of urban/rural settings, including two urban categories (urban conurbation, urban city and town) and two rural categories (rural town and fringe, rural village, hamlet and isolated dwelling).

### 2.5 Statistical analyses

To investigate the associations between local primary care services, satisfaction with health services and self-rated health, multilevel Poisson regression modelling was carried out to account for nested data structure and adjust for sociodemographic factors (age, sex, social class, education) and area level factors (deprivation, urban/rural settings). To examine whether the availability of GP practices was particularly important in deprived areas or rural areas, interaction terms between GP practices (0 vs 1+), deprivation quintiles and four types of urban/rural settings were fitted in the modelling. The missing data on social class and education were included as additional group in the analyses. Maximum likelihood estimation was used to account for missing data on satisfaction with health services and self-rated health based on the missing at random assumption. To compare the associations across the two waves, cross-sectional weights in Understanding Society were applied so the results were representative to the older populations in England at specific time points. A test for trend was used to examine whether higher availability of primary care services was associated with better health service satisfaction and self-rated health.

Sensitivity analyses were carried out to test whether different measures for primary care services would affect the main results. The availability of GP practices in neighbouring areas and average road distance to a GP surgery were included in the adjusted model including GP practice in local areas. All statistical analyses were conducted using Stata 15.1.

## 3 Results

Table 1 reports the characteristics of the study population at wave 3 (2011–13) and 6 (2014–16). Both waves had a higher percentage of younger age groups, women, urban residents and those from the least deprived settings. Most participants in the two waves had middle social class and no educational qualifications. In wave 3, 21.4% of participants reported fair or poor satisfaction with local health service and 27.0% had fair or poor self-rated health. In wave 6, the percentage of fair or poor satisfaction was 24.5% and 27.7% reported fair or poor health.

**Table 1:** Characteristics of study population at wave 3 and 6.

	Wave 3 (2011–2013)	Wave 6 (2014–2016)
Total	14,498	13,025

Age group		
50–54	2809 (19.4)	2455 (18.9)
55–59	2401 (16.6)	2199 (16.9)
60–64	2569 (17.7)	2037 (15.6)
65–69	2282 (15.7)	2193 (16.8)
70–74	1751 (12.1)	1596 (12.3)
75–79	1278 (8.8)	1193 (9.2)
80–84	870 (6.0)	782 (6.0)
85+	538 (3.7)	570 (4.4)

Sex		
Women	7946 (54.8)	7119 (54.7)
Men	6552 (45.2)	5906 (45.3)

Social class		
High (I/II)	4607 (31.8)	4457 (34.2)
Middle (III-NM/III-M)	5355 (36.9)	4822 (37.0)
Low (IV/VI)	2654 (18.3)	2318 (17.8)
Missing	1882 (13.0)	1428 (11.0)

Education		
Higher degree	3590 (24.8)	3652 (28.0)
Secondary	699 (4.8)	707 (5.4)
Primary	3077 (21.2)	2985 (22.9)
None of above	5251 (36.2)	4108 (31.5)
Missing	1881 (13.0)	1573 (12.1)

Urban/rural setting		
Urban conurbation	4819 (33.2)	4446 (34.1)
Urban city and town	6388 (44.1)	5623 (43.2)
Rural town and fringe	1581 (10.9)	1387 (10.7)
Rural village, hamlet and isolated dwelling	1710 (11.8)	1569 (12.1)

Deprivation 2015		
Q1 (Most)	2391 (16.5)	2055 (15.8)
Q2	2602 (18.0)	2314 (17.8)
Q3	3094 (21.3)	2799 (21.5)
Q4	3183 (22.0)	2920 (22.4)
Q5 (Least)	3228 (22.3)	2937 (22.6)

Number of GP practices		
0	11,696 (80.7)	10,501 (80.6)
1	2276 (15.7)	2065 (15.9)
2+	526 (3.6)	459 (3.5)

Number of public health/ community health service		
0	14,300 (98.6)	12,660 (97.2)
1+	198 (1.4)	365 (2.8)

Number of walk in centre/out of hour practices		
0	14,402 (99.3)	12,936 (99.3)
1+	96 (0.7)	89 (0.7)

**Fig. 1:**
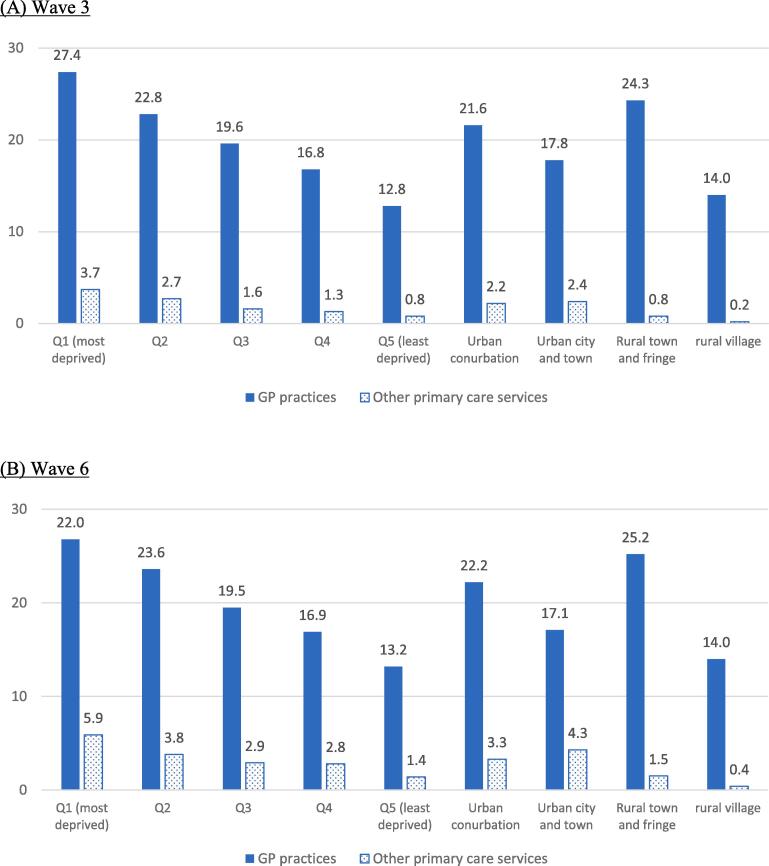
The percentages of participants who had at least one GP practices and other primary care services (walk in centres, out of hour practices, public health and community health services) in local areas by deprivation quintiles and urban/rural settings (%).

For both waves, approximately 80% of participants had no GP practices in their local areas while about 3.5% had 2 or more GP practices. The percentage of WIC/OOH practices was <1% and was similar across the two waves. For public health/community health services, the percentage was twice as high in wave 6 (2.8%) than wave 3 (1.4%). The percentage of participants who had at least one GP practices in local areas decreased from the most to least deprived quintile and this decreasing pattern was observed in both wave 3 (Fig. 1A) and wave 6 (Fig. 1B). Over 20% of the participants had at least one GP practices in urban conurbation areas and rural town and fringe areas. In urban city and town areas and rural village, hamlet and isolated dwelling areas, less than one-fifths had a local GP surgery. For other types of community-based primary care services, the percentage also decreased from the most to least deprived quintiles and was higher in urban than rural participants.

Table 2 shows the associations between the availability of local GP practices, satisfaction with health services and self-rated health. People who had two or more GP practices in local areas were less likely to report dissatisfaction with health services in both wave 3 (0.67; 95% CI: 0.52, 0.85) and wave 6 (0.74; 95% CI: 0.59, 0.92) than those who did not have local GP practices. The strength of associations remained similar when adjusting for sociodemographic and area level factors. For self-rated health, the unadjusted associations were not clear. After adjusting for individual and area level factors, people living in areas with two or more GP practices were less likely to report fair or poor health in wave 3 (0.80; 95% CI: 0.68, 0.96) compared to those who did not have GP practice in local areas. However, the association was not found in wave 6. The results of public health/community health services and WIC/OOH practices are reported in Table S1, Supporting Information. The associations were unclear for these services.

**Table 2:** Weighted results of the associations between the availiablity of GP practices in local areas, satisfaction with health services and self-rated health.

	Satisfaction with health services		Self-rated health	
	Wave 3 (2011–2013)	Wave 6 (2014–2016)	Wave 3 (2011–2013)	Wave 6 (2014–2016)
**Number of GP practices**	IRR (95% CI)	IRR (95% CI)	IRR (95% CI)	IRR (95% CI)
Model 1				
0	Ref.	Ref.	Ref.	Ref.
1	0.82 (0.73, 0.91)	0.90 (0.81, 1.00)	1.02 (0.93, 1.13)	1.13 (1.03, 1.23)
2+	0.70 (0.55, 0.89)	0.77 (0.62, 0.97)	1.01 (0.84, 1.21)	1.05 (0.87, 1.26)
Test for trends	<0.01	<0.01	0.71	0.03

Model 2				
0	Ref.	Ref.	Ref.	Ref.
1	0.81 (0.73, 0.91)	0.90 (0.81, 1.00)	1.01 (0.92, 1.11)	1.11 (1.03, 1.21)
2+	0.69 (0.54, 0.88)	0.76 (0.60, 0.95)	0.95 (0.79, 1.14)	0.99 (0.83, 1.18)
Test for trends	<0.01	<0.01	0.80	0.11

Model 3				
0	Ref.	Ref.	Ref.	Ref.
1	0.81 (0.72, 0.90)	0.89 (0.80, 0.99)	0.97 (0.88, 1.06)	1.06 (0.98, 1.16)
2+	0.67 (0.52, 0.85)	0.74 (0.59, 0.92)	0.80 (0.68, 0.96)	0.86 (0.72, 1.03)
Test for trends	<0.01	<0.01	0.03	0.82

Model 1: unadjsuted; Model 2: adjsuted for age, sex, social class and education; Model 3: adjsuted for age, sex, social class, education, deprivation and urban/rural settings.

Table 3 reports the results of the associations between the availability of local GP practices, satisfication with heatlh services and self-rated health by deprivation quintiles and urban/rural settings. For the satisfaction measure, the associations did not vary across urban/rural settings and deprivation quintiles. For self-rated health, people living in urban conurbation areas and the most deprived quintile were more likely to report fair/poor health than their counterparts across both waves. However, the associations between the availability of local GP practices and self-rated health were similar across urban/rural settings and deprivation quintiles.

**Table 3:** Weighted results of the associations between the avaliability of local GP practices (any vs none), satisfaction with health services and self-rated health by deprivation levels and urban/rural settings (adjusted for age, sex, social class and education).

	Satisfaction with health services		Self-rated health	
	Wave 3	Wave 6	Wave 3	Wave 6
**Urban/rural settings**	IRR (95% CI)	IRR (95% CI)	IRR (95% CI)	IRR (95% CI)
Urban conurbation	0.82 (0.68, 0.98)	0.92 (0.78, 1.09)	1.01 (0.88, 1.16)	1.04 (0.92, 1.19)
Urban city and town	0.80 (0.68, 0.92)	0.86 (0.74, 1.00)	0.96 (0.85, 1.08)	1.10 (0.97, 1.24)
Rural town and fringe	0.77 (0.57, 1.03)	0.90 (0.70, 1.17)	1.02 (0.78, 1.34)	0.99 (0.79, 1.25)
Rural villages	0.79 (0.57, 1.10)	0.80 (0.59, 1.09)	1.08 (0.78, 1.50)	1.32 (1.04, 1.68)
p-value for interaction	0.99	0.85	0.87	0.30

**Deprivation**				
Q1 (most)	0.70 (0.56, 0.88)	0.95 (0.77, 1.15)	0.94 (0.82, 1.08)	0.90 (0.78, 1.05)
Q2	0.85 (0.69, 1.05)	0.95 (0.77, 1.16)	0.88 (0.73, 1.07)	1.05 (0.89, 1.25)
Q3	0.77 (0.62, 1.05)	0.83 (0.67, 1.01)	0.95 (0.77, 1.16)	1.03 (0.88, 1.21)
Q4	0.69 (0.55, 0.88)	0.66 (0.51, 0.84)	1.00 (0.81, 1.24)	1.17 (0.97, 1.41)
Q5 (least)	0.89 (0.68, 1.17)	0.96 (0.75, 1.22)	0.93 (0.73, 1.18)	1.14 (0.90, 1.44)
p-value for interaction	0.49	0.12	0.94	0.23

The results of sensitivity analyses are reported in Table S2, Supporting Information. The availability of GP practices in neighbouring areas had weak associations with the satisfaction and self-rated health measures and did not affect the effect sizes of local GP practices. People living in areas with shorter distance to a GP surgery were less likely to report dissatisfaction with health services across the two waves but these associations were not found in self-rated health. Further including the distance measure did not change the associations for the availability of local GP practices.

## 4 Discussion

Using a nationwide cohort study of the general population in the UK, this study investigated the relationships between the availability of local primary care services, satisfaction with health services, self-rated health in older people at two time points. In both waves, older people who had one or more GP practices in their local areas were less likely to report dissatisfaction with health services than those who had none. However, the availability of local GP practices was not associated with self-rated health. These associations did not largely vary across urban/rural settings and deprivation levels.

### 4.1 Strengths and limitations

The data from UKHLS provide a representative sample including a large number of older people with different backgrounds across diverse regions of England. The design of panel survey and repeated measures across the follow-up waves allowed to compare cross-sectional associations at two time points. Based on the NHS Digital data, different types of primary care services were identified for the study areas and the information on open/close dates was used to determine the availability of services at specific time periods. In addition to self-rated health, this study included the satisfaction measure to identify potential barriers of using health services.

This study had some limitations. The quantity of primary care services was measured at LSOA level. Although LSOAs have been widely used in the UK census and small area statistics, the boundaries of this area unit might not reflect activity spaces of individuals (Weiss et al., 2007). People could travel outside of their local areas to access GP practices and other primary care services or use private healthcare services. However, the sensitivity analyses showed limited effects of GP practices in neighbouring areas. The number of primary care services only indicated the availability of relevant facilities in local areas but did not provide information on the staff size and experiences of visiting primary care services. Some surgeries might be understaffed and could not provide sufficient appointments for older adults. Other factors such as public transport, availability of appointments, travel time may also affect use of primary care services and consequently influence satisfaction and health conditions of individuals. Although these measures were not included in this study, adjustment for deprivation and urban/rural settings might partially account for the potential effect of these factors. Given the nature of cross-sectional analyses, the results may not imply causal direction. It is possible that older people living with chronic conditions and poor health moved to more deprived areas, which generally had higher availability of local primary care services. Although this study focused on those who continuously lived in the same addresses since the previous waves, participants might have moved before the study period. The study population was representative to the general population in England and the study weights were applied in the analyses. Yet the results might not be generalisable to other UK countries or regions due to variation in healthcare systems.

### 4.2 Comparison with existing literature

The results of this study echoed the previous empirical data (Adams and White, 2004, Jordan et al., 2004, Todd et al., 2014, Uk, 2015) and did not support the hypothesis of inverse care law (Tudor, 1971). In both waves, older people living in more deprived areas were found to have higher availability of all types of primary care services. Although socioeconomic disadvantage and area deprivation have been recognised as key determinants of multimorbidity and complex needs of healthcare (Ingram et al., 2021, Stafford et al., 2017), the spatial distribution of primary care services in England seems to correspond to the potential needs across deprivation levels. Yet this might not match the amount of care needed in highly deprived areas.

The availability of local primary care services was positively related to satisfaction with health services but not self-rated health in older people across the two waves. The presence of local GP practices may support older people to access healthcare in local areas and have a positive experience of primary care services. However, this seems to have limited effects on health. The results here differ from research on younger people, which shows strong relationships between poor experience of GP services (unhappy with explanation, not at ease with GPs, lack of respect by GPs and unable to talk about personal issues) and poor physical and mental health conditions (Yassaee et al., 2017). While older people generally report higher satisfaction with primary care services than younger age groups (Robertson et al., 2018), issues including restriction of only one health problem per appointment, difficulties of having the same GPs and continuity of care were reported in a focus group survey of people aged 65 or above from Healthwatch England (Healthwatch England, 2015). This may highlight the gap between the existing services and healthcare needs of older people, who are likely to live with multiple chronic conditions and require frequent and in-depth assessments. Although more GP practices and primary care facilities in local areas may provide more available appointments and improve satisfaction with the services, a primary and community care model is crucial to coordinate complex needs of health and social care and influence health and wellbeing in older age (World Health Organisation, 2019, Frost et al., 2020).

### 4.3 Implications for research and practice

This study suggests that the availability of local primary care services may have a positive impact on satisfaction with health services in older people but not their health. To optimise the supportive role of primary care services in healthy ageing, it is important to identify complex needs of health and social care in older people and their experience of using the services (Healthwatch England, 2015). In addition to the quantity, indicators related to the quality of care are needed to capture transition toward a community-based model of care (World Health Organisation, 2019, Frost et al., 2020).

Despite the large body of literature on health inequality and deprivation (Marmot et al., 2020), empirical evidence from this study did not support inverse care law. Older people living in deprived areas had higher availability of local primary care services but experienced worse health. To identify the opportunities for primary care services to support healthy ageing, future research should investigate healthcare needs of older people and develop possible interventions and care models in community-based settings (Abdi et al., 2019).

## Funding

This study was funded by UKRI-NHMRC Built Environment and Prevention Research Scheme ‘The impact of the Environment and Pollution On Cognitive Health (EPOCH): Building the knowledge base through international collaboration’ (MR/T038500/1). The funders had no roles in study design, data collection, analysis and interpretation of data, the writing of the report and the decision to submit the article for publication.

## Ethical approval

The University of Essex Ethics Committee has approved all data collection on Understanding Society main study and innovation panel waves, including asking consent for all data linkages except to health records. Requesting consent for health record linkage was approved at Wave 1 by the National Research Ethics Service (NRES) Oxfordshire REC A (08/H0604/124), at BHPS Wave 18 by the NRES Royal Free Hospital & Medical School (08/H0720/60) and at Wave 4 by NRES Southampton REC A (11/SC/0274). Approval for the collection of biosocial data by trained nurses in Waves 2 and 3 of the main survey was obtained from the National Research Ethics Service (Understanding Society - UK Household Longitudinal Study: A Biosocial Component, Oxfordshire A REC, Reference: 10/H0604/2).

## Data availability statement

Understanding Society: the UK Household Longitudinal Study is available via UK Data Service (https://beta.ukdataservice.ac.uk/datacatalogue/studies/study?id=6614). The prescribing centre data is available from the NHS Digital webpage (https://digital.nhs.uk/services/organisation-data-service/file-downloads/gp-and-gp-practice-related-data).

## Declaration of Competing Interest

The authors declare that they have no known competing financial interests or personal relationships that could have appeared to influence the work reported in this paper.
