# Supplementary material for: The availability of local primary care services, satisfaction with health services and self-rated health in older English adults: A population-based study
Source: Prev Med Rep. 2022 Apr 5;27:101786. doi: 10.1016/j.pmedr.2022.101786 (PMC9152809; doi:10.1016/j.pmedr.2022.101786)
Supplement: Supplementary Data 1 [file mmc1.docx]

**The availability of local primary care services, satisfaction with health services and self-rated health in older English adults: a population-based study**

**Supporting Information**

**Table S1.** Weighted results of the associations between the avaliability of public health/community health services, walk in centres/out of hour practices in local areas, satisfaction with health services and self-rated health

|  | Satisfaction with health services | | Self-rated health | |
| --- | --- | --- | --- | --- |
|  | Wave 3  (2011-2013) | Wave 6  (2014-2016) | Wave 3  (2011-2013) | Wave 6  (2014-2016) |
|  | IRR (95% CI) | IRR (95% CI) | IRR (95% CI) | IRR (95% CI) |
| **Number of public health/ community health services** | | | | |
| Model 1 |  |  |  |  |
| 0 | Ref. | Ref. | Ref. | Ref. |
| 1+ | 0.95 (0.65, 1.38) | 0.92 (0.74, 1.16) | 1.30 (1.02, 1.65) | 1.08 (0.89, 1.30) |
| p-value | 0.78 | 0.48 | 0.03 | 0.45 |
| Model 2 |  |  |  |  |
| 0 | Ref. | Ref. | Ref. | Ref. |
| 1+ | 0.96 (0.66, 1.39) | 0.91 (0.73, 1.14) | 1.23 (0.98, 1.56) | 1.03 (0.86, 1.25) |
| p-value | 0.81 | 0.43 | 0.08 | 0.72 |
| Model 3 |  |  |  |  |
| 0 | Ref. | Ref. | Ref. | Ref. |
| 1+ | 0.91 (0.62, 1.33) | 0.88 (0.70, 1.10) | 1.01 (0.79, 1.29) | 0.91 (0.75, 1.11) |
| p-value | 0.62 | 0.26 | 0.95 | 0.37 |
| **Number of walk in centres/ out of hour practices** | | | | |
| Model 1 |  |  |  |  |
| 0 | Ref. | Ref. | Ref. | Ref. |
| 1+ | 0.80 (0.46, 1.40) | 1.01 (0.63, 1.63) | 0.97 (0.62, 1.52) | 1.11 (0.76, 1.63) |
| p-value | 0.44 | 0.96 | 0.89 | 0.58 |
| Model 2 |  |  |  |  |
| 0 | Ref. | Ref. | Ref. | Ref. |
| 1+ | 0.80 (0.46, 1.42) | 1.01 (0.63, 1.63) | 0.90 (0.56, 1.43) | 0.99 (0.68, 1.44) |
| p-value | 0.45 | 0.96 | 0.65 | 0.96 |
| Model 3 |  |  |  |  |
| 0 | Ref. | Ref. | Ref. | Ref. |
| 1+ | 0.81 (0.46, 1.41) | 1.01 (0.63, 1.63) | 0.84 (0.54, 1.32) | 0.90 (0.62, 1.32) |
| p-value | 0.45 | 0.97 | 0.46 | 0.61 |

Model 1: unadjsuted; Model 2: adjsuted for age, sex, social class and education; Model 3: adjsuted for age, sex, social class, education, deprivation and urban/rural settings

**Table S2.** Sensitivity analyses: the number of GP practices in local LSOAs and neighbouring areas, average road distance to a GP surgery in LSOA (all models adjsuted for age, sex, social class, education, deprivation and urban/rural settings)

(A) Wave 3

|  | Model 1 | Model 2 | Model 3 | Model 4 |
| --- | --- | --- | --- | --- |
|  | IRR (95% CI) | IRR (95% CI) | IRR (95% CI) | IRR (95% CI) |
| **Satisfaction with health services** | | | | |
| Number of GP practices |  |  |  |  |
| *Local areas* |  |  |  |  |
| None | - | - |  | - |
| 1 | 0.81 (0.72, 0.90) | 0.80 (0.71, 0.89) |  | 0.88 (0.78, 0.99) |
| 2+ | 0.67 (0.52, 0.85) | 0.65 (0.51, 0.83) |  | 0.71 (0.55, 0.92) |
| *Neighbouring areas* |  |  |  |  |
| None |  | - |  |  |
| 1 |  | 0.95 (0.86, 1.03) |  |  |
| 2+ |  | 0.90 (0.82, 0.99) |  |  |
| Average road distance to a GP practice |  |  |  |  |
| ≥2 km |  |  | - | - |
| 1-2 km |  |  | 0.81 (0.73, 0.90) | 0.82 (0.74, 0.91) |
| 0.5-1 km |  |  | 0.70 (0.63, 0.79) | 0.74 (0.66, 0.84) |
| ≤0.5 km |  |  | 0.67 (0.57, 0.80) | 0.76 (0.63, 0.92) |
| **Self-rated health** | | | | |
| Number of GP practices |  |  |  |  |
| *Local areas* |  |  |  |  |
| None | - | - |  | - |
| 1 | 0.97 (0.88, 1.06) | 0.97 (0.88, 1.06) |  | 0.97 (0.88, 1.08) |
| 2+ | 0.80 (0.68, 0.96) | 0.80 (0.67, 0.95) |  | 0.80 (0.67, 0.96) |
| *Neighbouring areas* |  |  |  |  |
| None |  | - |  |  |
| 1 |  | 1.01 (0.93, 1.09) |  |  |
| 2+ |  | 0.98 (0.90, 1.06) |  |  |
| Average road distance to a GP practice |  |  |  |  |
| >2 km |  |  | - | - |
| 1-2 km |  |  | 0.95 (0.86, 1.05) | 0.95 (0.86, 1.05) |
| 0.5-1 km |  |  | 0.90 (0.81, 1.00) | 0.92 (0.82, 1.03) |
| ≤0.5 km |  |  | 0.94 (0.82, 1.09) | 0.99 (0.85, 1.16) |

(B) Wave 6

|  | Model 1 | Model 2 | Model 3 | Model 4 |
| --- | --- | --- | --- | --- |
|  | IRR (95% CI) | IRR (95% CI) | IRR (95% CI) | IRR (95% CI) |
| **Satisfaction with health services** | | | | |
| Number of GP practices |  |  |  |  |
| *Local areas* |  |  |  |  |
| None | - | - |  | - |
| 1 | 0.89 (0.80, 0.99) | 0.87 (0.79, 0.97) |  | 0.97 (0.86, 1.08) |
| 2+ | 0.74 (0.59, 0.92) | 0.72 (0.57, 0.90) |  | 0.79 (0.62, 1.01) |
| *Neighbouring areas* |  |  |  |  |
| None |  | - |  |  |
| 1 |  | 0.90 (0.83, 0.99) |  |  |
| 2+ |  | 0.86 (0.78, 0.94) |  |  |
| Average road distance to a GP practice |  |  |  |  |
| ≥2 km |  |  | - | - |
| 1-2 km |  |  | 0.78 (0.70, 0.86) | 0.78 (0.71, 0.87) |
| 0.5-1 km |  |  | 0.73 (0.65, 0.81) | 0.74 (0.66, 0.83) |
| ≤0.5 km |  |  | 0.69 (0.59, 0.82) | 0.73 (0.61, 0.88) |
| **Self-rated health** | | | | |
| Number of GP practices |  |  |  |  |
| *Local areas* |  |  |  |  |
| None | - | - |  | - |
| 1 | 1.06 (0.98, 1.16) | 1.05 (0.97, 1.15) |  | 1.11 (1.01, 1.22) |
| 2+ | 0.86 (0.72, 1.03) | 0.85 (0.71, 1.01) |  | 0.90 (0.75, 1.09) |
| *Neighbouring areas* |  |  |  |  |
| None |  | - |  |  |
| 1 |  | 0.93 (0.85, 1.00) |  |  |
| 2+ |  | 0.93 (0.86, 1.01) |  |  |
| Average road distance to a GP practice |  |  |  |  |
| >2 km |  |  | - | - |
| 1-2 km |  |  | 1.06 (0.96, 1.17) | 1.05 (0.95, 1.16) |
| 0.5-1 km |  |  | 1.00 (0.90, 1.11) | 0.98 (0.88, 1.09) |
| ≤0.5 km |  |  | 0.95 (0.83, 1.10) | 0.92 (0.78, 1.07) |
